# Description of *Cohnella rhizoplanae* sp. nov., isolated from the root surface of soybean (*Glycine max*)

**DOI:** 10.1007/s10482-024-02051-y

**Published:** 2024-12-24

**Authors:** Peter Kämpfer, Stefanie P. Glaeser, John A. McInroy, Hans-Jürgen Busse, Dominique Clermont, Alexis Criscuolo

**Affiliations:** 1 Institut für Angewandte Mikrobiologie, Justus-Liebig-Universität Giessen, Heinrich-Buff-Ring 26–32, 35392 Giessen, Germany; 2 Division – Entomology and Plant Pathology Dept., Auburn University, Alabama, USA; 3 Division of Clinical Microbiology and Infection Biology, Institut Für Bakteriologie, Mykologie Und Hygiene, Veterinärmedizinische Universität, Vienna, Austria; 4 Institut Pasteur, Université de Paris, CIP - Collection of Institut Pasteur, 75015 Paris, France; 5 GIPhy - Genome Informatics and Phylogenetics, Biological Resource Center of Institut Pasteur, Institut Pasteur, Université de Paris, 75015 Paris, France

**Keywords:** *Cohnella*, Taxonomy, Phylogenetics, Genomics

## Abstract

A Gram-staining-positive, aerobic bacterium, designated strain JJ-181^ T^, was isolated from the root surface of soybean. Based on the 16S rRNA gene sequence similarities, strain JJ-181^ T^ was grouped into the genus *Cohnella*, most closely related to *Cohnella hashimotonis* F6_2S_P_1^T^ (98.85%) and *C. ginsengisoli* GR21-5^ T^ (98.3%). The pairwise average nucleotide identity and digital DNA-DNA hybridisation values of the JJ-181^ T^ genome assembly against publicly available *Cohnella* type strain genomes were below 84% and 28%, respectively. The fatty acid profile from whole cell hydrolysates, the cell wall diaminoacid, the quinone system, the polar lipid profile, and the polyamine pattern supported the allocation of strain JJ-181^ T^ to the genus *Cohnella*. In addition, the results of physiological and biochemical tests also allowed phenotypic differentiation of strain JJ-181^ T^ from its closely-related *Cohnella* species. Functional analysis revealed that strain JJ-181^ T^ has different gene clusters related to swarming motility, chemotaxis ability, and endospore and biofilm formations. The gene content also suggests the ability of strain JJ-181^ T^ to synthesise biotin and riboflavin, as well as indole-3-acetic acid, an important phytohormone for plant growth. Based on polyphasic analyses, strain JJ-181^ T^ can be classified as a new species of the genus *Cohnella*, for which we propose the name *Cohnella rhizoplanae* sp. nov., with strain JJ-181^ T^ (= LMG 31678^ T^ = CIP 112018^ T^ = CCM 9031^ T^ = DSM 110650^ T^) as the type strain.

**Supplementary Information:**

The online version contains supplementary material available at 10.1007/s10482-024-02051-y.

## Introduction

The genus *Cohnella* was initially described as a homogeneous group within the family *Paenibacillaceae* (Kämpfer et al. 2006). At present the genus *Cohnella* is composed of 36 species with validated names (https://lpsn.dsmz.de/genus/cohnella), including the type species *C. thermotolerans* (Kämpfer et al. 2006). There are also 10 putative additional species, for which the names have not been validated so far. All members of this genus can be differentiated from those of the genus *Paenibacillus* on the basis of 16S rRNA gene sequence analysis, the polar lipid patterns and also the fatty acid compositions. As the major menaquinone MK-7 is detected, major fatty acids are *iso*-C_16:0_, *anteiso*-C_15:0_ and C_16:0_ and the predominant polar lipids are diphosphatidylglycerol, phosphatidylglycerol and phosphatidylethanolamine (Kämpfer et al. 2006).

In contrast to other plant growth-promoting bacteria (e.g. members of *Bacillus* or *Paenibacillus*), *Cohnella* is a poorly studied genus, from a plant-beneficial function contributing point of view. However, most of the type strains were isolated from different soils (Cai et al. 2010; Khianngam et al. 2010a,b; Kim et al. 2010, 2011; Yoon et al. 2007; Jiang et al. 2012; Wang et al. 2012; Yoon and Jung 2012), whereas some were isolated from the rhizosphere (Hameed et al. 2013) or endophytic compartments and root nodules of different host plants (Flores-Félix et al. 2014; García-Fraile et al. 2008). Furthermore, cultivation-independent molecular studies indicated a substantial role of *Cohnella* spp. in soils, especially in association with plants. For example, a high relative abundance of *Cohnella* species sequences in 16S rRNA gene amplicon data generated after DNA-based stable isotope probing (SIP) using ^13^C-labelled root exudates indicated that this taxon plays an important role in the top and subsoil rhizosphere microbiome (Uksa et al. 2017).

Here, we describe a new strain, designated as JJ-181^ T^ and isolated from the root surface (rhizoplane) of soybean (*Glycine max*) grown near Dunbar, Nebraska USA. Functional analyses suggest that strain JJ-181^ T^ could be an efficient root coloniser, with dedicated functionalities such as swarming motility, chemotaxis ability, sporulation capacity and/or biofilm formation. Moreover, its gene content also suggests that strain JJ-181^ T^ could have the ability to produce biotin, riboflavin, paeninodin-related peptides and indole-3-acetic acid. Finally, polyphasic taxonomy results assess that strain JJ-181^ T^ should be assigned to a novel species of the genus *Cohnella*, for which the name *Cohnella rhizoplanae* sp. nov. is proposed, with JJ-181^ T^ (= LMG 31678^ T^ = CIP 112018^ T^ = CCM 9031^ T^ = DSM 110650^ T^) as the type strain.

## Materials and methods

### Isolation, culture conditions and physiological tests

In 2014, a Gram-staining-positive bacterial strain, designated JJ-181^ T^ was isolated from the root surface of soybean (*Glycine max*) grown near Dunbar, Nebraska USA (GPS coordinates: 40.653060, –96.067579). Isolation was carried out as reported previously (Kämpfer et al. 2017). The bacterium was subcultivated on tryptone soy agar (TSA; Oxoid), at 28 °C for 24 h. Gram-staining was performed as described previously by Gerhardt et al. (1994) after three days of culture. All cell morphological traits were observed under a Zeiss light microscope at a magnification of ⋅1000, using cells that had been grown for three days at 28 °C on TSA (Oxoid).

Temperature-dependent growth was tested on nutrient agar at 4, 10, 15, 20, 25, 28, 30, 36, 45, 50, and 55 °C. NaCl tolerance was investigated at different concentrations of NaCl (0.5, 1.0, 2.0, 3.0, …, 8.0 w/v %) in tryptic soy broth (TSB). The pH dependent growth was tested in TSB adjusted with HCl and NaOH to pH values between 4.0 and 12.0.

The strain was physiologically and biochemically characterised using the 96-well plate test system (Kämpfer et al. 1991) and by some additional biochemical tests: production of hydrogen sulphide using the lead acetate paper and triple-sugar-iron methods, indole reaction with Ehrlich’s and Kovacs’ reagents, activity of arginine dihydrolase, lysine decarboxylase, ornithine decarboxylase, DNase (Oxoid CM321; supplemented with 0.01% toluidine blue), β-galactosidase (ONPG), urease on Christensen’s urea agar (Kämpfer 1990), hydrolysis of casein, gelatin (plate method), starch, and tyrosine (Smibert & Krieg 1994).

### 16S rRNA gene sequencing and analyses

The 16S rRNA gene of strain JJ-181^ T^ (grown on TSA) was PCR-amplified with the primer system Eub9f and Eub1492R (5´-GAGTTTGATCMTGGCTCAG-3´ and 5´-ACGGYTACCTTGTTACGACTT-3´, respectively; Lane 1991) from a cell lysate according to Schauss et al. (2015). Sanger sequencing was performed with primers Eub9f and E786F (5´-GATTAGATACCCTGGTAG-3´). MEGA11 v11.0.10 (Tamura et al. 2021) was used to manually correct and assemble the sequence.

BLASTn search against the 16S rRNA RefSeq database of curated type strain sequences (accessed 2024/06/23) was carried out for a first phylogenetic identification. Detailed phylogenetic analysis including all type strains of currently described *Cohnella* species was performed in ARB release 5.2 (Ludwig et al. 2004) with the “All-Species Living Tree" Project (LTP; Yarza et al. 2008) database (release LTP_04_2021, September 2021; Ludwig et al. 2021). Sequences not present in the database were imported and aligned in the alignment explorer generated for the respective database. The sequence was added to the database tree using the parsimony quick ad marked tool of ARB and the gap95_q0_to_q5 filter as recommended by Ludwig et al. (2021).

The multiple sequence alignment was checked manually based on secondary structure information. Pairwise nucleotide sequence identities were calculated in ARB. Phylogenetic classifications were carried out from the multiple sequence alignment restricted to positions 98 to 1423 (according to *Escherichia coli* numbering; Brosius et al. 1978). Phylogenetic trees were inferred with the maximum likelihood criterion using RAxML v7.04 (Stamatakis 2006) with GTR-GAMMA and rapid bootstrap analysis (100 replicates), the neighbour-joining method (ARB Neighbour-joining) with the Jukes and Cantor (1969) correction, and the maximum parsimony criterion using DNAPARS v3.6 (https://phylipweb.github.io/phylip/doc/dnapars.html).

### Genome sequencing and analyses

The whole-genome sequencing of strain JJ-181^ T^ (grown on TSA) was carried out with a NextSeq500 apparatus using the Nextera XT DNA library preparation kit (Illumina) and a 2 × 150 bp paired-end protocol, yielding 2,589,483 read pairs (114 × sequencing depth and 385 bp insert size, on average). Read processing and genome assembly were performed using fq2dna v21.06 (https://gitlab.pasteur.fr/GIPhy/fq2dna). Gene prediction and annotation of the assembled scaffolds were performed by the NCBI Prokaryotic Genome Annotation Pipeline (PGAP; Li et al. 2021) when included in the RefSeq repository under the accession number NZ_CAOJCN010000000.

Completeness and contamination indices of the whole genome assembly were estimated using CheckM v1.1.3 (Parks et al. 2015). A consensus 16S rRNA segment was independently assembled using ASSU v1.1 (https://gitlab.pasteur.fr/GIPhy/ASSU) to assess genome sequence authenticity.

Plant-beneficial function contributing (PBFC) genes were searched using BLASTp against the predicted coding sequences (CDS) of JJ-181^ T^. Biosynthetic gene clusters for secondary metabolites were inferred using antiSMASH v7.0.0beta1-67b538a9 (Blin et al. 2023).

Pairwise average nucleotide and amino acid identity (ANI and AAI, respectively) values were computed using OGRI_B v1.2 (https://gitlab.pasteur.fr/GIPhy/OGRI) between the draft genome of JJ-181^ T^ and every publicly available *Cohnella* type strain genome.

A pan-genome analysis was performed by clustering the predicted CDS from the JJ-181^ T^ and other *Cohnella* type strain genomes into orthologous families using OrthoFinder v2.5.5 (Emms and Kelly 2019). The CDS were classified into functional clusters of orthologous groups (COG) categories using COGniz v1.1 (https://gitlab.pasteur.fr/GIPhy/COGniz) with the 2020 update of the COG database (Galperin et al. 2021).

A phylogenetic classification of these genomes was inferred using JolyTree v2.1 (Criscuolo 2019, 2020). A phylogenetic tree was also inferred using the 120 universal single-copy genes suggested by Parks et al. (2017). Gene sequences were searched using tBLASTn against the genome sequences. For each locus, gathered amino acid sequences were aligned using MAFFT v7.505 (Katoh and Standley 2013, 2016), and the aligned characters were processed using BMGE v2.0 (Criscuolo and Gribaldo 2010) to select those suited for phylogenetic analysis. Maximum likelihood phylogenetic inference from the concatenation of the 120 resulting multiple sequence alignments (44,384 aligned characters) was carried out using IQ-TREE v2.3.4 (Minh et al. 2020) with evolutionary model LG + F + R5 (derived by minimising the Bayesian information criterion) and UFboot branch supports (1,000 replicates).

### Chemotaxonomy

For the detection of the diagnostic diamino acid of the cell wall, biomass that had been grown at 28 °C in 3.3xPYE broth (1.0 g peptone from casein, 1.0 g yeast extract, pH 7.2) for three days was used. The detection was carried out as described by Schumann (2011). Polyamines, quinones and polar lipids were also extracted from biomass that had been grown in 3.3xPYE broth. Biomass subjected to polyamine analysis was harvested at the late exponential growth phase, as recommended by Busse and Auling (1988), whereas quinones and polar lipids were extracted from cells harvested at the stationary growth phase. Extraction of polyamines was carried out as described by Busse and Auling (1988), applying HPLC conditions reported by Busse et al. (1997). For extraction and analyses of quinones and polar lipids, the integrated procedures reported by Tindall (1990a, b) and Altenburger et al. (1996) were applied. HPLC equipment was reported by Stolz et al. (2007). The polyamine pattern consisted of 32.0 µmol (g dry weight)^−1^ spermidine, 2.7 µmol (g dry weight)^−1^ spermine, 0.5 µmol (g dry weight)^−1^ putrescine and 0.1 µmol (g dry weight)^−1^ caderverine.

Fatty acid analysis was carried out as described by Kämpfer and Kroppenstedt (1996) using a HP-6890 gas chromatograph, Sherlock MIDI software version 2.11, and a TSBA peak naming table version 4.1. Strains were cultivated on R2A agar at 28 °C for 48 h prior to extraction.

## Results and discussion

### 16S rRNA gene sequence

The sequenced 16S rRNA gene sequence of strain JJ-181^ T^ (accession: OP288081) is a continuous stretch of 1,392 unambiguous nucleotides. Analyses of the 16S rRNA sequence show that JJ-181^ T^ shares the highest 16S rRNA gene sequence similarity (e.g. 94.2% to 98.3%) with the members of the genus *Cohnella*, the closely-related ones being the type strains of *C. hashimotonis* (98.85%) and *C. ginsengisoli* (98.3%); all other nucleotide similarities were below 98%. According to the phylogenetic trees inferred from the 16S rRNA gene sequences, strain JJ-181^ T^ forms a distinct clade together with the type strains of *C. hashimotonis, C. ginsengisoli, C. rhizosphaerae,* “*C. plantaginis* “ and “*C. capsica*” (Fig. S1).

### Genome features

The draft genome of JJ-181^ T^ is made of 6,781,507 bps on 71 contigs (N50, 246,382), with 60.48% G + C content. Genome sequence authenticity was assessed by aligning the 16S rRNA segment derived from Sanger sequencing (accession: OP288081) against the one assembled from the sequencing reads using BLASTn, leading to > 99.4% pairwise sequence similarity. Completeness and contamination indices of the genome assembly are 99.73% and 0.43%, respectively.

Average nucleotide and amino acid identity (ANI and AAI, respectively) values against publicly available *Cohnella* type strain genomes were reported in Table S1, together with the associated digital DNA-DNA hybridisation (dDDH) values (formula 2; https://ggdc.dsmz.de). All these estimates are far below the commonly admitted species delineation cutoffs, e.g. ANI, 95%; AAI, 96%; (d) DDH, 70%.

**Fig. 1:**
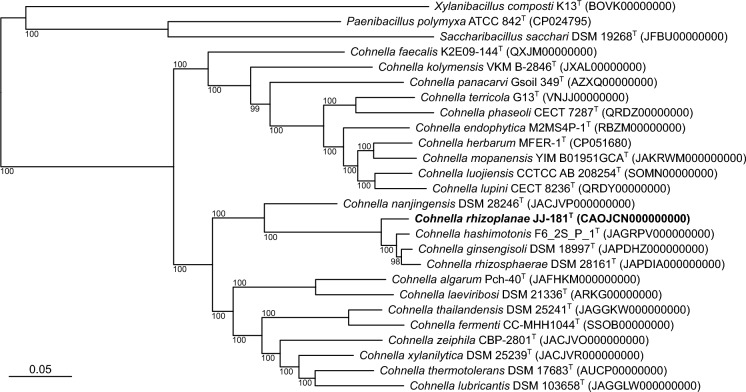
Phylogenetic tree showing the phylogenetic relationship of strain JJ-181^ T^ to species of the genus *Cohnella*. The type species of *Paenibacillus*, *Saccharibacillus* and *Xylanibacillus* were used as an outgroup. Numbers at branch nodes refer to UFboot values (1,000 replicates). Bar, 0.05 amino acid substitutions per aligned character

Phylogenetic classifications based on whole genomes (Fig. S2) and multiple conserved genes (Fig. 1) both confirmed that strain JJ-181^ T^ is a member of the genus *Cohnella*. They placed strain JJ-181^ T^ into a well-supported clade (100% branch supports) containing the three type strains of *C. ginsengisoli, C. hashimotonis* and *C. rhizosphaerae* (the so-called *C. ginsengisoli* clade, according to Simpson et al. 2023), in accordance with the JJ-181^ T^ phylogenetic neighbouring derived from the 16S rRNA gene sequences (Fig. S1).

Annotation of the JJ-181^ T^ genome assembly predicted a total of 5,661 coding sequences (CDS), in coherence with the observed CDS content within *Cohnella* type strain genomes (Fig. S3). A total of 3,612 CDS were classified into 23 COG categories, of which category G (Carbohydrate transport and metabolism) constituted the maximal assigned one (568), followed by category K (Transcription; 394). The distribution of the assigned COG categories for the JJ-181^ T^ predicted CDS is comparable to the ones observed within the *Cohnella* genus (Fig. S3). Pan-genome analysis of the *Cohnella* type strain genomes revealed that 16.1% (911) of the JJ-181^ T^ predicted CDS fall within the (strict) core, whereas 3.3% (187) are putatively unique (almost all being annotated as hypothetical protein). Of note, a large set of putatively unique CDS was observed to be concentrated into a specific nucleotide segment (corresponding to accessions WP_271751533-WP_271751583) which seemed unique within the *Paenibacillaceae* genomes (according to BLASTn searches; not shown).

Different CDS potentially related to root colonisation were observed (Table S2), such as those related to chemotaxis ability (e.g. gene cluster *cheBAWCD* and different methyl-accepting chemotaxis protein) and swarming motility (of which a cluster of 25 *flg*/*fli* genes). Interestingly, the JJ-181^ T^ genome was found to also contain three clusters of genes that bear some amino-acid similarity with the ones making up the *pep*-2 cluster in the genome of *Paenibacillus polymyxa* WLY78 (Table S2); this cluster seems to be involved in exopolysaccharide biosynthesis and biofilm formation (He et al. 2021). Moreover, different CDS related to quorum sensing were also observed (Table S2). This brings up the hypothesis that JJ-181^ T^ has the ability to form biofilm, a key determinant of root colonisation (e.g. Knights et al. 2021). Of note, although microscopical analysis did not show any endospore formation (see below), many related genes were also observed, e.g. up to 56 CDS associated to the GO process “asexual sporulation” (GO:0030436), some being regrouped into gene clusters (e.g. *spoIIIA*, *spoVA*; not shown).

Some other genes potentially promoting plant growth were also observed (Table S3), in particular those related to phosphate transport, such as the operon *phoU*-*pstBACS* comparable to those shared by some *Paenibacillus* species (e.g. Xie et al. 2016; Yuan et al. 2022); however, virtually no genes related to phosphate solubilisation (e.g. *phn* genes) were found. A set of genes related to the production of indole-3-acetic acid (IAA, an important phytohormone that enhances plant growth and development, e.g. Spaepen et al. 2007) was also observed, such as the operon *trpABCDE* (Table S3). Of note, very few CDS were found that are related to nitrogen fixation (e.g. *nif* genes) and to nitrate/nitrite transport and reduction (e.g. *nar* and *nir* genes), suggesting that strain JJ-181^ T^ does not have these abilities (just as the other species from the *C. ginsengisoli* clade; Simpson et al. 2023). Interestingly, the genome of JJ-181^ T^ was also found to share a cluster of six *bio* genes, as well as seven *fab* genes (Table S3); as these different genes are constituting a biotin biosynthesis pathway (e.g. Ma et al. 2024), this suggests that JJ-181^ T^ has the ability to synthesise this B-group vitamin to support plant growth when required (e.g. Palacios et al. 2014; Shameer and Prasad 2018). Genes corresponding to a complete riboflavin pathway were also found (Table S3), suggesting that JJ-181^ T^ is also capable of synthesising riboflavin, another plant growth stimulator (e.g. Rao 1973).

Finally, six biosynthetic gene clusters (BGCs) involved in secondary metabolite production were predicted (Table S4). Two of these BGCs were labelled as lasso peptides (often involved in antimicrobial activities, e.g. Hegemann et al. 2015) and showed some similarity to the (five-gene) paeninodin BGC described for *Paenibacillus dendritiformis* C454 (Zhu et al. 2016); interestingly, based on our pan-genome analysis, one of these two lasso peptide BGCs were also found (with important gene-content similarity, assessed using Jaccard index > 40%) in nine other *Cohnella* type strain genomes, whereas the second one was observable in only the three *C. ginsengisoli* clade genomes, as well as in the phylogenetically distant *C. faecalis* type strain genome (Table S4). Three other predicted BGCs were also observed in the two type strain genomes of *C. ginsengisoli* and *C. hashimotonis*: one type III polyketide synthase (T3PKS), one agrD-like cyclic lactone autoinducer peptide, and one proteusin (Table S4). Finally, the sixth predicted BGC, a non-ribosomal peptide synthetase fragment (NRPS), seemed specific to JJ-181^ T^.

### Morphological, physiological and biochemical specifications

Strain JJ-181^ T^ was observed to grow well on nutrient agar, tryptone-soy (TS) agar, and R2A agar (all Oxoid), Columbia agar supplement with 5% sheep blood and 3.3xPYE agar at 28 °C after 48 h; no haemolysis was observed on blood agar. No growth was observed on MacConkey agar. The strain was observed to grow well in a temperature range from 20 to 36 °C; no growth was observed at 15 °C (and below) and at 45 °C (and above). When a suspension of cells in 3.3 × PYE broth was incubated for 15 min at 80 °C and afterwards incubated overnight at 28 °C, unambiguous growth was demonstrated by strongly increased turbidity. These observations demonstrate that cells of strain JJ-181^ T^ either tolerate high temperatures or grow as the result of germinated endospores (as suggested by the functional analysis; see above). However, light microscopical analysis of strain JJ-181^ T^ at 1000-fold magnification did not show endospore formation after growth on TS agar at 28 °C for 48 h. The O/F test for glucose was negative. In the S.I.M test, no production of H_2_S or indole was observed. Salinity-dependent growth was tested in TS broth (Oxoid) by the addition of 1, 2, and 3% (v/w%) NaCl. Strain JJ-181^ T^ was observed to grow without NaCl, and in the presence of 1% and 2% NaCl. The pH range of growth (tested in TS broth adjusted to pH 4.5 to 12.5) ranged from pH 4.5 to 9.5; no growth was observed at pH values 4.0 and 10.5.

The results of physiological characterisation, performed using methods described previously (Kämpfer 1990; Kämpfer et al. 1991), were reported in the species description (see also Table 1). After 72 h of incubation at 25 °C, strain JJ-181^ T^ was observed to be only able to produce acids from D-glucose, but not from any other sugars or sugar-related compounds; however, it was observed to be able to utilise several of them (weakly) as sole sources of carbon. A distinct physiological and biochemical profile allowed differentiation of the strain from the type strains of the two most closely-related species.

**Table 1:** Differential phenotypic characteristics between strain JJ-181^ T^ (1) in comparison with *Cohnella rhizosphaerae* CSE 5610^ T^ (2), *C. hashimotonis* DSM 115098^ T^ (3), *C. plantaginis* DSM 25424^ T^ (4), *C. ginsengisoli* DSM 18997^ T^ (5) and *C. thermotolerans* CCUG 47242^ T^ (6). Data (1- 5) are from this study; data in parentheses are from Wang et al. (2012); data (6) are from Kämpfer et al. (2006)

Characteristics	1	2	3	4	5	6
Growth temperature (°C)	20 − 40	15 − 45	15 − 37	10 − 45 (10 − 45)	10 − 36 (10 − 40)	20 − 54
Growth pH	4.5 − 9.5	5.5 − 9.5	5.5 − 8	4.5 − 8.5 (5.0 − 8.0)	4.5 − 8.5 (6.0 − 9.0)	n.d
Growth in the presence of 3% NaCl	−	+	+	+ ( +)	( −)	n.d
Nitrate reduction	−	−	W	− ( −)	( +)	n.d
Assimilation of:						
Alanine	−	−	+	− ( +)	− ( −)	−
Histidine	−	−	+	− ( +)	− ( −)	−
Serine	−	−	−	− ( +)	− ( −)	−
Sucrose	+	W	+	− ( +)	− ( −)	−
L − Arabinose	+	W	+	− ( −)	W ( +)	W
Salicin	+	−	W	− ( −)	W ( +)	−
Melibiose	+	W	+	− ( −)	W ( +)	+
D − Fructose	−	W	+	− ( +)	W ( −)	+

+ , positive reaction; −, negative reaction; W, weak positive reaction; n.d., not determined

### Chemotaxonomic characteristics

The quinone system was found to contain menaquinones MK-7 (99.8%) and MK-8 (0.2%). Diphosphatidylglycerol was identified as the major polar lipid. In addition, high proportions of phosphatidylethanolamine, phosphatidylglycerol, two unidentified aminophospholipids and an unidentified phospholipid were only detectable after total lipid staining, as well as small amounts of two unidentified lipids (Fig. S1). Lysyl-phosphatidylglycerol was found absent. Like the presence of *meso-*diaminopimelic acid as the diagnostic diamino acid of the peptidoglycan and the major quinone MK-7, this polar lipid profile conforms well to the description of the genus (Kämpfer et al. 2006; García-Fraile et al. 2008; Khianngam et al. 2010b). Strain JJ-181^ T^ is the first member of the genus *Cohnella* analysed for its polyamine content, but the fact that it was resembling those of other endospore-formers (Hamana et al. 1989) suggests that this diagnostic feature is of minor importance with this group of bacteria.

The derived fatty acid profile was observed to comprise mainly *iso*- and *anteiso*-branched fatty acids, which is similar to the most closely-related *Cohnella* species. The detailed fatty acid profile obtained from cells grown on R2A-medium after 48 h incubation at 28 °C were reported in Table S5.

## Conclusion

Phylogenetic analyses assessed taxonomic placement of strain JJ-181^ T^ within the genus *Cohnella,* and the quinone system as well as the fatty acid profile were found to be in accordance with this assignment*.* ANI, AAI and dDDH analyses to closely-related *Cohnella* type strain genomes clearly showed that strain JJ-181^ T^ represents a novel species for which we propose the name *Cohnella rhizoplanae* sp. nov., with JJ-181^ T^ (= LMG 31678^ T^ = CIP 112018^ T^ = CCM 9031^ T^ = DSM 110650^ T^) as the type strain.

### Description of *Cohnella rhizoplanae* sp. nov.

(rhi.zo.pla´nae. Gr. n. rhiza a root; L. neut. n. *planum*, flat ground, surface; N.L. fem. n. *rhizoplana* the rhizoplane; N.L. gen. n. *rhizoplanae*, of the rhizoplane, the region of the root epidermis of a plant where soil particles and bacteria adhere).

Cells are Gram-staining-positive, strictly aerobic rods (0.8–1.0 µm in diameter, 2.0–3.0 µm in length). Motility and endospore formation were not detected. Colonies grown on R2A agar are circular, convex and beige. Optimal temperature for growth is 28 °C; growth occurs at 20–36 °C but not at 15 °C and 45 °C. Optimal pH for growth in TS broth is pH 6.5; growth occurs at pH 4.5–9.5 and in TS broth containing NaCl concentrations up to 2% (w/v). Test for catalase is negative, oxidase activity is positive. Tests for urease, gelatinase, arginine dihydrolase, lysine decarboxylase, ornithine decarboxylase, tryptophan deaminase and citrate utilisation are negative. Starch, casein and gelatin are hydrolysed. Indole production, H_2_S formation, DNase, and the Voges-Proskauer reaction are also negative. Acid formation from sugars could only be observed for D-glucose, but not with the following compounds: D-xylose, lactose, sucrose, D-mannitol, dulcitol, salicin, D-adonitol, i-inositol, D-sorbitol, L-arabinose, raffinose, L-rhamnose, maltose, trehalose, cellobiose, erythritol, melibiose and D-arabitol. Only a few compounds were utilised as a sole source of carbon by strain JJ-181^ T^, among them: N-acetyl-D-glucosamine, L-arabinose, D-cellobiose, D-glucose, D-maltose, D-mannose, D-melibiose, ribose, sucrose, salicin, D-trehalose, D-xylose, D-maltitol, D-mannitol.

The compounds D-adonitol, arbutin, D-fructose, D-galactose, gluconate, L-rhamnose, m-inositol, D-sorbitol, malate, pyruvate, putrescine, acetate, propionate, cis-aconitate, trans-aconitate, adipate, 4-aminobutyrate, azelate, citrate, itaconate, 2-oxoglutarate, alanine, histidine, serine, and mesaconate are not utilised as sole carbon source.

*Meso*-diaminopimelic acid is the cell wall diaminoacid. The quinone system contains predominantly menaquinone MK-7. In the polar lipid profile, diphosphatidylglycerol is predominant and high amounts of phosphatidylglycerol, phosphatidylethanolamine, two unidentified aminophospholipids and one phospholipid are present as well. Lysyl-phosphatidylglycerol is absent. The polyamine pattern contains the major compound spermidine. Major fatty acids are *iso*-C_15:0_, and *iso*-C_16:0_ and *anteiso*-C_15:0_. The G + C content of the genomic DNA is 60.48%.

The type strain JJ-181^ T^ (= LMG 31678^ T^ = CIP 112018^ T^ = CCM 9031^ T^ = DSM 110650^ T^) was isolated from the root surface of soybean (*Glycine max*) grown near Dunbar, Nebraska USA (GPS coordinates: 40.653060, – 96.067579). The genome sequence of strain JJ-181^ T^ is available under the GenBank/EMBL/DDBJ accession number CAOJCN000000000 and the Sanger-sequenced 16S rRNA gene sequence under OP288081. The current RefSeq accession number for the whole genome sequence is NZ_CAOJCN010000000.

## Supplementary Information

Below is the link to the electronic supplementary material.

Supplementary file1 (PDF 960 KB)

## Data Availability

The raw sequencing reads were deposited in the European Nucleotide Archive (ENA) repository under the BioProject accession number PRJEB55565 (run accession number ERR10784662). The 16S rRNA gene sequence is available under the GenBank/EMBL/DDBJ accession number OP288081. The genome assembly is available under the GenBank/EMBL/DDBJ accession number CAOJCN000000000.
