# Supplementary material for: Description of Cohnella rhizoplanae sp. nov., isolated from the root surface of soybean (Glycine max)
Source: Antonie Van Leeuwenhoek. 2024 Dec 24;118(2):41. doi: 10.1007/s10482-024-02051-y (PMC11668882; doi:10.1007/s10482-024-02051-y)

Antonie van Leeuwenhoek

Cohnella rhizoplanae sp. nov., isolated from the root surface of soybean (*Glycine max*)

Peter Kämpfer [1,*], Stefanie P. Glaeser [1], John A. McInroy [2], Hans-Jürgen Busse [3], Dominique Clermont [4], Alexis Criscuolo [5]

[1] Institut für Angewandte Mikrobiologie, Justus-Liebig-Universität Giessen, D-35392 Giessen, Germany

[2] Auburn University, Alabama, USA

[3] Division of Clinical Microbiology and Infection Biology, Institut für Bakteriologie, Mykologie und Hygiene, Veterinärmedizinische Universität, Wien, Austria

[4] Institut Pasteur, Université de Paris, CIP - Collection of Institut Pasteur, F-75015 Paris, France

[5] Institut Pasteur, Université de Paris, GIPhy - Genome Informatics and Phylogenetics, Biological Resource Center of Institut Pasteur, F-75015 Paris, France

[*] Corresponding author (peter.kaempfer@umwelt.uni-giessen.de)

Supplementary Data

**Table S1**	Overall genome relatedness indices (ANI, AAI, dDDH)
**Tables S2-S3**	Putative plant-beneficial function contributing genes
**Table S4**	Gene clusters for secondary metabolites
**Table S5**	Cellular fatty acid profiles
**Figure S1**	16S rRNA phylogenetic tree
**Figure S2**	Genome-based phylogenetic tree
**Figure S3**	COG analysis
**Figure S4**	Polar lipid profiles

Table S1. Overall genome relatedness indices between strain JJ-181^{T} and next related *Cohnella* type strains

type strain	genome accession	%ANI [CI]	%AAI [CI]	%dDDH [CI]
C. ginsengisoli DSM 18997^{T}	JAPDHZ000000000	83.95 [83.76-84.12]	86.74 [86.43-87.07]	27.70 [25.3-30.2]
C. hashimotonis F6_2S_P_1^{T}	JAGRPV000000000	83.44 [83.25-83.62]	85.98 [85.64-86.27]	26.90 [24.6-29.4]
C. rhizosphaerae DSM 28161^{T}	JAPDIA000000000	83.24 [83.01-83.44]	85.69 [85.29-86.02]	27.20 [24.9-29.7]
C. nanjingensis DSM 28246^{T}	JACJVP000000000	75.34 [75.05-75.65]	72.16 [71.62-72.55]	21.40 [19.2-23.9]
C. xylanilytica DSM 25239^{T}	JACJVR000000000	74.46 [74.20-74.71]	70.15 [69.72-70.57]	20.80 [18.5-23.2]
C. thermotolerans DSM 17683^{T}	AUCP00000000	73.93 [73.67-74.19]	70.77 [70.27-71.15]	20.00 [17.8-22.4]
C. zeiphila CBP-2801^{T}	JACJVO000000000	73.60 [73.35-73.87]	69.33 [68.91-69.77]	20.20 [18.0-22.7]
C. lubricantis DSM 103658^{T}	JAGGLW000000000	73.52 [73.25-73.76]	69.80 [69.24-70.30]	19.50 [17.3-21.9]
C. laeviribosi DSM 21336^{T}	ARKG00000000	73.26 [72.97-73.52]	70.12 [69.60-70.64]	19.50 [17.3-21.9]
C. fermenti CC-MHH1044^{T}	SSOB00000000	73.22 [72.95-73.53]	68.60 [68.14-69.11]	20.60 [18.4-23.1]
C. thailandensis DSM 25241^{T}	JAGGKW000000000	73.03 [72.75-73.29]	69.04 [68.56-69.54]	20.00 [17.8-22.4]
C. algarum Pch-40^{T}	JAFHKM000000000	72.90 [72.61-73.16]	69.08 [68.54-69.59]	19.90 [17.7-22.3]
C. panacarvi Gsoil 349^{T}	AZXQ00000000	72.10 [71.82-72.35]	67.60 [67.13-68.05]	19.60 [17.4-22.0]
C. faecalis K2E09-144^{T}	QXJM00000000	71.35 [71.13-71.63]	68.15 [67.71-68.72]	20.60 [18.4-23.0]
C. phaseoli CECT 7287^{T}	QRDZ00000000	71.06 [70.78-71.32]	66.75 [66.29-67.20]	19.40 [17.2-21.8]
C. terricola G13^{T}	VNJJ00000000	70.92 [70.61-71.23]	67.55 [67.03-68.02]	19.90 [17.7-22.3]
C. endophytica M2MS4P-1^{T}	RBZM00000000	70.73 [70.46-71.04]	67.45 [66.96-67.99]	19.00 [16.9-21.4]
C. lupini CECT 8236^{T}	QRDY00000000	70.47 [70.19-70.73]	67.56 [67.10-67.95]	20.60 [18.3-23.0]
C. kolymensis VKM B-2846^{T}	JXAL00000000	70.31 [70.03-70.57]	68.35 [67.85-68.98]	18.80 [16.6-21.2]
C. luojiensis CCTCC AB 208254^{T}	SOMN00000000	70.17 [69.87-70.46]	67.68 [67.13-68.24]	18.50 [16.4-20.9]
C. herbarum MFER-1^{T}	CP051680	69.92 [69.62-70.20]	66.85 [66.38-67.29]	21.50 [19.2-23.9]
C. mopanensis YIM B01951GCA^{T}	JAKRWM000000000	69.75 [69.49-70.05]	67.43 [66.93-67.91]	19.20 [17.0-21.6]

Table S2. Genes of strain JJ-181^{T} potentially related with root colonization

accession	gene	annotated product	putative function
			**Swarming motility**
WP_271749295	rpoD	RNA polymerase sigma factor RpoD	
WP_271749577	flgB	flagellar basal body rod protein FlgB	
WP_271749578	flgC	flagellar basal body rod protein FlgC	
WP_271749579	fliE	flagellar hook-basal body complex protein FliE	
WP_271749580	fliF	flagellar basal-body MS-ring/collar protein FliF	
WP_271749581	fliG	flagellar motor switch protein FliG	
WP_271749583	-	FliH/SctL family protein	
WP_271749584	fliI	flagellar protein export ATPase FliI	
WP_271749923	fliJ	flagellar export protein FliJ	
WP_271749585	-	MgtE protein	
WP_271749586	-	flagellar hook-length control protein FliK	
WP_271749587	flgD	flagellar hook assembly protein FlgD	
WP_271749588	-	TIGR02530 family flagellar biosynthesis protein	
WP_271749589	flgG	flagellar basal body rod protein FlgG	
WP_271749590	-	flagellar FlbD family protein	
WP_271749591	-	flagellar basal body-associated FliL family	
WP_271749592	fliM	flagellar motor switch protein FliM	
WP_271749593	fliY	flagellar motor switch phosphatase FliY	
WP_090117377	-	response regulator	
WP_271749596	-	flagellar biosynthetic protein FliO	
WP_271749597	fliP	flagellar type III secretion system pore protein FliP	
WP_271749598	fliQ	flagellar biosynthetic protein FliQ	
WP_271749600	fliR	flagellar biosynthetic protein FliR	
WP_271749924	flhB	flagellar biosynthesis protein FlhB	
WP_271749601	flhA	flagellar biosynthesis protein FlhA	
WP_271749602	flhF	flagellar biosynthesis protein FlhF	
WP_271749610	-	FliA/WhiG family RNA polymerase sigma factor	
WP_271752125	rpoD	RNA polymerase sigma factor RpoD	
WP_271752705	-	flagellar hook-basal body protein	
WP_271752706	-	flagellar hook-basal body protein	
WP_271753027	motA	flagellar motor stator protein Mot	
WP_271753029	-	flagellar motor protein MotB	
WP_271753649	-	flagellar protein	
WP_334219837	flgM	flagellar biosynthesis anti-sigma factor FlgM	
WP_271753652	-	flagellar protein FlgN	
WP_271753653	flgK	flagellar hook-associated protein FlgK	
WP_271753654	flgL	flagellar hook-associated protein FlgL	
WP_271753655	-	DUF6470 family protein	
WP_271753656	fliW	flagellar assembly protein FliW	
WP_271753657	csrA	carbon storage regulator CsrA	
WP_271753659	-	flagellin	
WP_271753678	fliD	flagellar filament capping protein FliD	
WP_271753772	fliS	flagellar export chaperone FliS	
WP_271753729	-	flagellar motor protein MotB	
WP_271753773	-	flagellar motor protein	

Table S2. (cont.)

accession	gene	annotated product	putative function
			**Chemotaxis ability**
WP_271749133	-	methyl-accepting chemotaxis protein	
WP_090117377	-	response regulator	
WP_271749604	-	chemotaxis protein CheB	
WP_271749605	-	chemotaxis protein CheA	
WP_271749606	-	chemotaxis protein CheW	
WP_271749607	-	chemotaxis protein CheC	
WP_271749608	-	chemotaxis protein CheD	
WP_271750081	-	HAMP domain-containing methyl-accepting chemotaxis protein	
WP_271750246	-	chemotaxis protein CheX	
WP_271750420	-	methyl-accepting chemotaxis protein	
WP_271750751	-	methyl-accepting chemotaxis protein	
WP_271750771	-	CHASE3 domain-containing protein	
WP_271751144	yycI	two-component system regulatory protein YycI	
WP_271751145	yycH	two-component system activity regulator YycH	
WP_271751621	-	chemotaxis protein CheX	
WP_271751622	-	response regulator	
WP_271751623	-	methyl-accepting chemotaxis protein	
WP_271751624	-	chemotaxis protein CheW	
WP_271751625	-	chemotaxis protein CheA	
WP_271751626	-	methyl-accepting chemotaxis protein	
WP_271751627	-	protein-glutamate O-methyltransferase CheR	
WP_271751628	-	chemotaxis response regulator protein-glutamate methylesterase	
WP_271751643	-	methyl-accepting chemotaxis protein	
WP_271751712	-	CheR family methyltransferase	
WP_271751816	-	methyl-accepting chemotaxis protein	
WP_271751869	-	methyl-accepting chemotaxis protein	
WP_271752031	-	protein-glutamate O-methyltransferase CheR	
WP_271752178	-	protein-glutamate O-methyltransferase CheR	
WP_271752339	-	protein-glutamate O-methyltransferase CheR	
WP_271753597	-	HAMP domain-containing methyl-accepting chemotaxis protein	
WP_271753957	-	methyl-accepting chemotaxis protein	
WP_271754037	-	methyl-accepting chemotaxis protein	
WP_271754254	-	HAMP domain-containing methyl-accepting chemotaxis protein	
WP_271754332	-	methyl-accepting chemotaxis protein	
WP_271754458	-	methyl-accepting chemotaxis protein	
WP_271754547	-	methyl-accepting chemotaxis protein	
WP_271754733	-	methyl-accepting chemotaxis protein	

Table S2. (cont.)

accession	gene	annotated product	putative function
			**Biofilm formation**
WP_271749610	-	FliA/WhiG family RNA polymerase sigma factor	
WP_271749915	-	YlbF family regulator	
WP_271749990	-	CpsD/CapB family tyrosine-protein kinase	
WP_271749992	galU	UTP--glucose-1-phosphate uridylyltransferase GalU	
WP_271749993	-	sugar transferase	
WP_271749994	-	UDP-glucose/GDP-mannose dehydrogenase family protein	
WP_271749995	-	glycosyltransferase family 4 protein	
WP_271749997	-	O-antigen ligase family protein	
WP_271749998	-	glycosyltransferase	
WP_271750000	-	oligosaccharide flippase family protein	
WP_271750002	-	glycosyltransferase	
WP_271750004	-	glycosyltransferase family 4 protein	
WP_271750006	-	glycosyltransferase family 4 protein	
WP_271750008	-	CatB-related O-acetyltransferase	
WP_271750010	-	CatB-related O-acetyltransferase	
WP_271750012	-	glycosyltransferase family 4 protein	
WP_271750014	-	glycosyltransferase	
WP_271750016	-	sugar phosphate nucleotidyltransferase	
WP_271750284	wecB	UDP-N-acetylglucosamine 2-epimerase (non-hydrolyzing)	
WP_271750753	-	glucose-1-phosphate adenylyltransferase	
WP_271750754	glgD	glucose-1-phosphate adenylyltransferase subunit GlgD	
WP_271750756	glgA	glycogen synthase GlgA	
WP_090116868	-	extracellular matrix/biofilm biosynthesis regulator RemA family protein	
WP_271751731	-	Wzz/FepE/Etk N-terminal domain-containing protein	
WP_271751732	-	glycosyltransferase	
WP_271751733	-	CpsD/CapB family tyrosine-protein kinase	
WP_271751734	-	sugar transferase	
WP_271751735	-	glycosyltransferase family 4 protein	
WP_271751736	-	UDP-glucose/GDP-mannose dehydrogenase family protein	
WP_271751737	-	O-antigen polymerase	
WP_271751738	-	glycosyltransferase family 1 protein	
WP_271751739	-	glycosyltransferase	
WP_271751740	-	MOP flippase family protein	
WP_271751741	galU	UTP--glucose-1-phosphate uridylyltransferase GalU	
WP_271751742	-	sugar phosphate nucleotidyltransferase	
WP_271752686	wecB	UDP-N-acetylglucosamine 2-epimerase (non-hydrolyzing)	
WP_271754009	cysE	serine O-acetyltransferase	
WP_271754406	-	type VI secretion system tube protein Hcp	
WP_271754826	-	Wzz/FepE/Etk N-terminal domain-containing protein	
WP_271754828	-	CpsD/CapB family tyrosine-protein kinase	
WP_271754830	galU	UTP--glucose-1-phosphate uridylyltransferase GalU	
WP_271754832	-	sugar transferase	
WP_271754834	-	glycosyltransferase	
WP_271754835	-	O-antigen ligase family protein	
WP_271754837	-	glycosyltransferase family 4 protein	
WP_271754839	-	glycosyltransferase	
WP_271754841	-	glycosyltransferase	
WP_271754843	-	oligosaccharide flippase family protein	
WP_271754845	-	acyltransferase	
WP_271754847	-	polysaccharide pyruvyl transferase family protein	
WP_271754849	-	sugar phosphate nucleotidyltransferase	
WP_271754851	-	UDP-glucose/GDP-mannose dehydrogenase family protein	

Table S2. (cont.)

accession	gene	annotated product	putative function
			**Quorum sensing**
WP_271749193	-	long-chain fatty acid--CoA ligase	
WP_271749513	ftsY	signal recognition particle-docking protein FtsY	
WP_271749558	ffh	signal recognition particle protein	
WP_271749792	-	DMT family transporter	
WP_271749793	-	DMT family transporter	
WP_271750806	spo0A	sporulation transcription factor Spo0A	
WP_271750855	ribD	bifunctional diaminohydroxyphosphoribosylaminopyrimidine deaminase/5-amino-6-(5-phosphoribosylamino)uracil reductase RibD	
WP_271751201	-	YidC/Oxa1 family membrane protein insertase	
WP_271751530	secG	preprotein translocase subunit SecG	
WP_271752345	trpE	anthranilate synthase component I	
WP_271752452	hfq	RNA chaperone Hfq	
WP_271752864	yajC	preprotein translocase subunit YajC	
WP_271752870	secD	protein translocase subunit SecD	
WP_271753685	secA	preprotein translocase subunit SecA	
WP_271753944	-	polyprenyl synthetase family protein	
WP_271754014	secE	preprotein translocase subunit SecE	
WP_271754057	secY	preprotein translocase subunit SecY	
WP_271754140	-	oligopeptide/dipeptide ABC transporter ATP-binding protein	
WP_271754141	-	ABC transporter ATP-binding protein	
WP_271754142	-	ABC transporter permease	
WP_271754143	-	ABC transporter permease	
WP_271754144	-	ABC transporter substrate-binding protein	

Table S3. Genes of strain JJ-181^T potentially related with plant growth-promoting traits

accession	gene	annotated product	putative function
WP_013373004	-	NifU family protein	**Nitrogen fixation**
WP_271750422	-	ammonium transporter	**Nitrate / nitrite transport / reduction**
WP_27175058	-	FAD-dependent oxidoreductase	
WP_271750707	-	formate/nitrite transporter family protein	
WP_271752918	-	ammonium transporter	
WP_271755051	nirB	nitrite reductase large subunit NirB	
WP_271755052	nirD	nitrite reductase small subunit NirD	
WP_271755053	-	formate/nitrite transporter family protein	
WP_271753516	-	alkaline phosphatase	**Phosphate solubilisation**
WP_271749082	-	phosphate ABC transporter substrate-binding protein	**Phosphate transporter**
WP_271749083	pstC	phosphate ABC transporter permease subunit PstC	
WP_271749084	pstA	phosphate ABC transporter permease PstA	
WP_271749085	pstB	phosphate ABC transporter ATP-binding protein PstB	
WP_271750500	-	ATP-binding protein	
WP_271750501	-	response regulator transcription factor	
WP_271751812	phoU	phosphate signaling complex protein PhoU	
WP_271751932	pstB	phosphate ABC transporter ATP-binding protein PstB	
WP_271751933	pstB	phosphate ABC transporter ATP-binding protein PstB	
WP_271751813	pstA	phosphate ABC transporter permease PstA	
WP_271751814	pstC	phosphate ABC transporter permease subunit PstC	
WP_271751815	-	phosphate ABC transporter substrate-binding protein PstS family protein	
WP_271750121	trpS	tryptophan--tRNA ligase	**Indole-3-acetic acid (IAA) production**
WP_271750434	-	3-deoxy-7-phosphoheptulonate synthase	
WP_271752341	aroC	chorismate synthase	
WP_271752342	aroB	3-dehydroquinate synthase	
WP_271752344	aroH	chorismate mutase	
WP_271752345	trpE	anthranilate synthase component I	
WP_271752346	trpD	anthranilate phosphoribosyltransferase	
WP_271752347	trpC	indole-3-glycerol phosphate synthase TrpC	
WP_271752348	--	phosphoribosylanthranilate isomerase	
WP_271752349	trpB	tryptophan synthase subunit beta	
WP_271752350	trpA	tryptophan synthase subunit alpha	

Table S3. (cont.)

accession	gene	annotated product	putative function
			**Biotin biosynthesis**
WP_271749061	fabV	enoyl-ACP reductase FabV	
WP_271749508	fabD	ACP S-malonyltransferase	
WP_271749509	fabG	3-oxoacyl-[acyl-carrier-protein] reductase	
WP_090117455	acpP	acyl carrier protein	
WP_271749510	fabF	beta-ketoacyl-ACP synthase II	
WP_271750710	fabI	enoyl-ACP reductase FabI	
WP_271752618	fabF	beta-ketoacyl-ACP synthase II	
WP_271752709	fabZ	3-hydroxyacyl-ACP dehydratase FabZ	
WP_271755230	bioC	malonyl-ACP O-methyltransferase BioC	
WP_271755231	-	alpha/beta fold hydrolase	
WP_271755232	bioF	8-amino-7-oxononanoate synthase	
WP_271755233	bioB	biotin synthase BioB	
WP_271755234	bioD	dethiobiotin synthase	
WP_271755240	bioA	adenosylmethionine--8-amino-7-oxononanoate transaminase	
			**Riboflavin biosynthesis**
WP_271749638	-	bifunctional riboflavin kinase/FAD synthetase	
WP_271750144	-	Cof-type HAD-IIB family hydrolase	
WP_271750855	ribD	bifunctional diaminohydroxyphosphoribosylaminopyrimidine deaminase/5-amino-6-(5-phosphoribosylamino)uracil reductase RibD	
WP_271750856	-	riboflavin synthase	
WP_271750857	-	bifunctional 3,4-dihydroxy-2-butanone-4-phosphate synthase/GTP cyclohydrolase II	
WP_217592710	ribE	6,7-dimethyl-8-ribityllumazine synthase	

Table S4. Biosynthetic gene clusters of strain JJ-181^{T} for secondary metabolites

accession	gene	annotated product	type
			**Type III polyketide synthase (T3PKS)**<br><br>Note: similar gene clusters also found in the genomes of *Cohnella ginsengisoli* DSM 18997^{T} (gene-content Jaccard index, 41.51%) and *C. hashimotonis* F6_2S_P_1^{T} (50.00%)
WP_271749795	-	cupin domain-containing protein	
WP_271749796	-	DUF1796 family putative cysteine peptidase	
WP_271749797	-	-	
WP_271749798	-	FMN-dependent NADH-azoreductase	
WP_271749799	-	MFS transporter	
WP_271749800	-	MarR family transcriptional regulator	
WP_271749801	rnz	Ribonuclease Z	
WP_271749802	selD	selenide, water dikinase SelD	
WP_271749803	mnmH	tRNA 2-selenouridine(34) synthase MnmH	
WP_271749804	-	AraC family transcriptional regulator	
WP_271749805	-	proline dehydrogenase family protein	
WP_271749806	-	glucose 1-dehydrogenase	
WP_271749807		TetR/AcrR family transcriptional regulator	
WP_271749808	-	discoidin domain-containing protein	
WP_271749809	-	Nif3-like dinuclear metal center hexameric protein	
WP_271749811	-	tetratricopeptide repeat protein	
WP_271749812	-	diaminopimelate dehydrogenase	
WP_271749813	-	-	
WP_271749814	-	type III polyketide synthase	
WP_271749815	-	NAD(P)/FAD-dependent oxidoreductase	
WP_271749817	-	glycerol-3-phosphate responsive antiterminator	
WP_271749818	glpK	glycerol kinase GlpK	
WP_271749937	-	FAD-dependent oxidoreductase	
WP_271749819	-	metallophosphoesterase	
WP_271749820	-	YdiU family protein	
WP_271749821	-	potassium channel family protein	
WP_271749822	-	glucose-6-phosphate isomerase	
WP_271749823	-	MBL fold metallo-hydrolase	
WP_271749824	-	Gfo/Idh/MocA family oxidoreductase	
WP_271749825		DUF3231 family protein	
WP_271749826	-	DUF2512 family protein	
WP_271749828	-	iron-sulfur cluster assembly protein	
WP_271749829	-	DinB family protein	
WP_271749830	-	WYL domain-containing protein	
WP_271749831	-	antibiotic biosynthesis monooxygenase	
WP_271749832	-	penicillin-binding transpeptidase domain-containing protein	
			**Lasso peptide**<br><br>Note: WP_271749983-8 similar to BGC0001356, i.e. paeninodin biosynthetic gene cluster from *Paenibacillus dendritiformis* C454<br><br>Note : similar gene clusters also found in the genomes of *Cohnella faecalis* K2E09-144^{T} (gene-content Jaccard index, 68.18%), *C. ginsengisoli* DSM 18997^{T} (61.54%), *C. hashimotonis* F6_2S_P_1^{T} (100.0%) and *C. rhizosphaerae* DSM 28161^{T} (94.44%)
WP_271749980	-	helix-turn-helix transcriptional regulator	
WP_271749981	-	glycosyl hydrolase family 28-related protein	
WP_271749982	-	-	
WP_271749983	-	asparagine synthase-related protein	
WP_110468933	-	paeninodin family lasso peptide	
WP_271749984	-	aldolase	
WP_271749985	-	lasso peptide biosynthesis PqqD family chaperone	
WP_271749986	-	lasso peptide biosynthesis B2 protein	
WP_271749987	-	nucleotidyltransferase family protein	
WP_271749988	-	ABC transporter ATP-binding protein	
WP_271749989	-	Wzz/FepE/Etk N-terminal domain-containing protein	
WP_271749990	-	CpsD/CapB family tyrosine-protein kinase	
WP_271749992	galU	UTP--glucose-1-phosphate uridylyltransferase GalU	
WP_271749993	-	sugar transferase	
WP_271749994	-	UDP-glucose/GDP-mannose dehydrogenase family protein	
WP_271749995	-	glycosyltransferase family 4 protein	

Table S4. (cont.)

accession	gene	annotated product	type
WP_271752533	-	sugar ABC transporter substrate-binding protein	**agrD-like cyclic lactone autoinducer peptides**<br><br>Note: similar gene clusters also found in the genomes of *Cohnella ginsengisoli* DSM 18997^T (gene-content Jaccard index, 43.75%) and *C. hashimotonis* F6_2S_P_1^T (86.95%)
WP_271752323	-	sugar ABC transporter ATP-binding protein	
WP_271752324	-	-	
WP_090112863	-	HU family DNA-binding protein	
WP_271752325	-	-	
WP_271752326	-	accessory gene regulator B family protein	
WP_271752327	-	cyclic lactone autoinducer peptide	
WP_271752328	-	LytTR family DNA-binding domain-containing protein	
WP_271752330	mtrB	trp RNA-binding attenuation protein MtrB	
WP_271752331	-	-	
WP_271752332	-	heptaprenyl diphosphate synthase component 1	
WP_271752333	-	demethylmenaquinone methyltransferase	
WP_271752334	-	UbiA-like polyprenyltransferase	
WP_271752335	-	flavin prenyltransferase UbiX	
WP_271752336	-	menaquinone biosynthesis protein	
WP_271752337	-	polyprenyl synthetase family protein	
WP_271752338	ndk	nucleoside-diphosphate kinase	
WP_271752339	-	protein-glutamate O-methyltransferase CheR	
WP_271752340	-	-	
WP_271752341	aroC	chorismate synthase	
WP_271753584	-	glycosyltransferase	**Non-ribosomal peptide synthetase fragment (NRPS)**
WP_271753585	-	cation diffusion facilitator family transporter	
WP_271753587	-	ABC transporter substrate-binding protein	
WP_271753589	-	ABC transporter permease subunit	
WP_271753590	-	carbohydrate ABC transporter permease	
WP_271753591	-	glycosyl hydrolase family 8	
WP_271753592	-	alpha-glucuronidase family glycosyl hydrolase	
WP_271753593	-	endo-1,4-beta-xylanase	
WP_271753594	-	MFS transporter	
WP_271753595	-	SH3 domain-containing C40 family peptidase	
WP_271753596	-	non-ribosomal peptide synthetase	
WP_271753597	-	HAMP domain-containing methyl-accepting chemotaxis protein	
WP_271753599	-	S-layer homology domain-containing protein	
WP_271753626	-	AraC family transcriptional regulator	
WP_271753600	-	glycoside hydrolase family 4	
WP_271753601	-	VOC family protein	
WP_271754535	-	MFS transporter	**Proteusin**<br><br>Note: similar gene clusters also found in the genomes of *Cohnella ginsengisoli* DSM 18997^T (gene-content Jaccard index, 46.15%) and *C. hashimotonis* F6_2S_P_1^T (88.23%)
WP_271754536	-	PAS domain S-box protein	
WP_271754537	-	zinc ribbon domain-containing protein	
WP_271754539	-	YrdB family protein	
WP_271754541	-	GNAT family N-acetyltransferase	
WP_271754542	-	fused MFS/spermidine synthase	
WP_271754543	-	-	
WP_271754544	-	NHLP leader peptide family RiPP precursor	
WP_271754545	-	HAMP domain-containing sensor histidine kinase	
WP_271754546	-	MerR family transcriptional regulator	
WP_271754547	-	methyl-accepting chemotaxis protein	
WP_271754548	-	HD-GYP domain-containing protein	
WP_271754549	-	aldo/keto reductase	
WP_271754551	-	MFS transporter	
WP_217593381	-	winged helix-turn-helix transcriptional regulator	
WP_271754552	rlmN	23S rRNA (adenine(2503)-C(2))-methyltransferase RlmN	

Table S4. (cont.)

accession	gene	annotated product	type
			**Lasso peptide**
WP_271754845	-	acyltransferase	Note: WP_271754856-65 similar to BGC0001356, i.e. paeninodin biosynthetic gene cluster from *Paenibacillus dendritiformis* C454<br><br>Note : similar gene clusters also found in the 9 genomes of *Cohnella faecalis* K2E09-144^{T} (gene-content Jaccard index, 63.64%), *C. fermenti* CC-MHH1044^{T} (50.00%), *C. ginsengisoli* DSM 18997^{T} (59.26%), *C. hashimotonis* F6_2S_P_1^{T} (60.00%), *C. nanjingensis* DSM 28246^{T} (62.50%), *C. rhizosphaerae* DSM 28161^{T} (70.83%), *C. thailandensis* DSM 25241^{T} (46.43%), *C. thermotolerans* DSM 17683^{T} (51.85%) and *C. zeiphila* CBP-2801^{T} (53.84%)
WP_271754847	-	polysaccharide pyruvyl transferase family protein	
WP_271754849	-	sugar phosphate nucleotidyltransferase	
WP_271754851	-	UDP-glucose/GDP-mannose dehydrogenase family protein	
WP_271754852	-	chitobiase/beta-hexosaminidase C-terminal domain-containing protein	
WP_271754854	-	sulfotransferase	
WP_271754856	-	asparagine synthase-related protein	
WP_110468926	-	paeninodin family lasso peptide	
WP_271754859	-	aldolase	
WP_271754860	-	lasso peptide biosynthesis PqqD family chaperone	
WP_271754862	-	lasso peptide biosynthesis B2 protein	
WP_271754863	-	nucleotidyltransferase family protein	
WP_271754865	-	ABC transporter ATP-binding protein	
WP_271754876	cysC	adenylyl-sulfate kinase	
WP_271754867	-	VanZ family protein	
WP_271754869	-	-	
WP_271754871	-	-	
WP_271754873	-	tyrosine-type recombinase/integrase	
WP_271754874	-	-	

Table S5. Fatty acid content of strain JJ-181^{T} (1) in comparison with *Cohnella rhizosphaerae* CSE 5610^{T} (2), *C. hashimotonis* F6_2S_P_1^{T} (3), *C. plantaginis* DSM 25424^{T} (4), *C. ginsengisoli* DSM 18997^{T} (5) and *C. thermotolerans* CCUG 47242^{T} (6). Data (1-2) and (4-5) are from this study; data (3) are from Simpson *et al.* (2023; doi:10.3389/fmicb.2023.1166013); data in parentheses are from Wang *et al.* (2012; doi:10.1007/s10482-012-9715-1); data (6) are from Kämpfer *et al.* (2006; doi:10.1099/ijs.0.63985-0). Values are percentages of the total fatty acids; -, not detected.

fatty acid	1	2	3	4	5	6
**saturated**						
$C_{12:0}$	-	3.3	-	5.7 (-)	3.6 (-)	-
$C_{14:0}$	-	5.6	2.0	- (1.6)	7.3 (1.6)	1.0
$C_{15:0}$	-	-	-	-	-	1.4
$C_{16:0}$	10.0	25.4	4.9	14.7 (7.7)	36.5 (9.6)	6.6
$C_{18:0}$	2.0	-	-	-	-	-
**branched**						
iso-$C_{13:0}$	11.3	-	-	-	-	-
iso-$C_{14:0}$	2.4	-	-	- (4.1)	- (6.6)	2.1
iso-$C_{15:0}$	20.9	10.2	10.5	11.7 (11.3)	6.6 (13.9)	3.2
iso-$C_{16:0}$	-	12.2	23.1	15.2 (18.6)	10.6 (21.6)	45.5
iso-$C_{17:0}$	-	-	1.1	- (1.2)	- (1.7)	-
anteiso-$C_{13:0}$	9.4	-	-	-	-	-
anteiso-$C_{15:0}$	5.4	36.5	51.7	43.8 (44.3)	30.2 (37.2)	28.4
anteiso-$C_{17:0}$	4.7	6.7	2.2	8.9 (3.1)	5.3 (2.8)	6.7
**unsaturated**						
$C_{18:1}\omega 6c$	-	-	-	-	-	4
$C_{18:1}\omega 9c$	-	-	1.1	- (1.4)	- (2.8)	-

Figure S1. Maximum likelihood tree based on 16S rRNA gene sequences showing the phylogenetic relationship of strain JJ-181^T to species of the genus *Cohnella*. Three *Paenibacillus* type strains were used as an outgroup. The sequence accession is specified between parentheses next after each taxon name. Numbers at branch nodes refer to bootstrap values >70% (100 replicates). Nodes marked with circles were supported by the maximum parsimony tree. Bar, 0.10 substitutions per nucleotide sequence position.

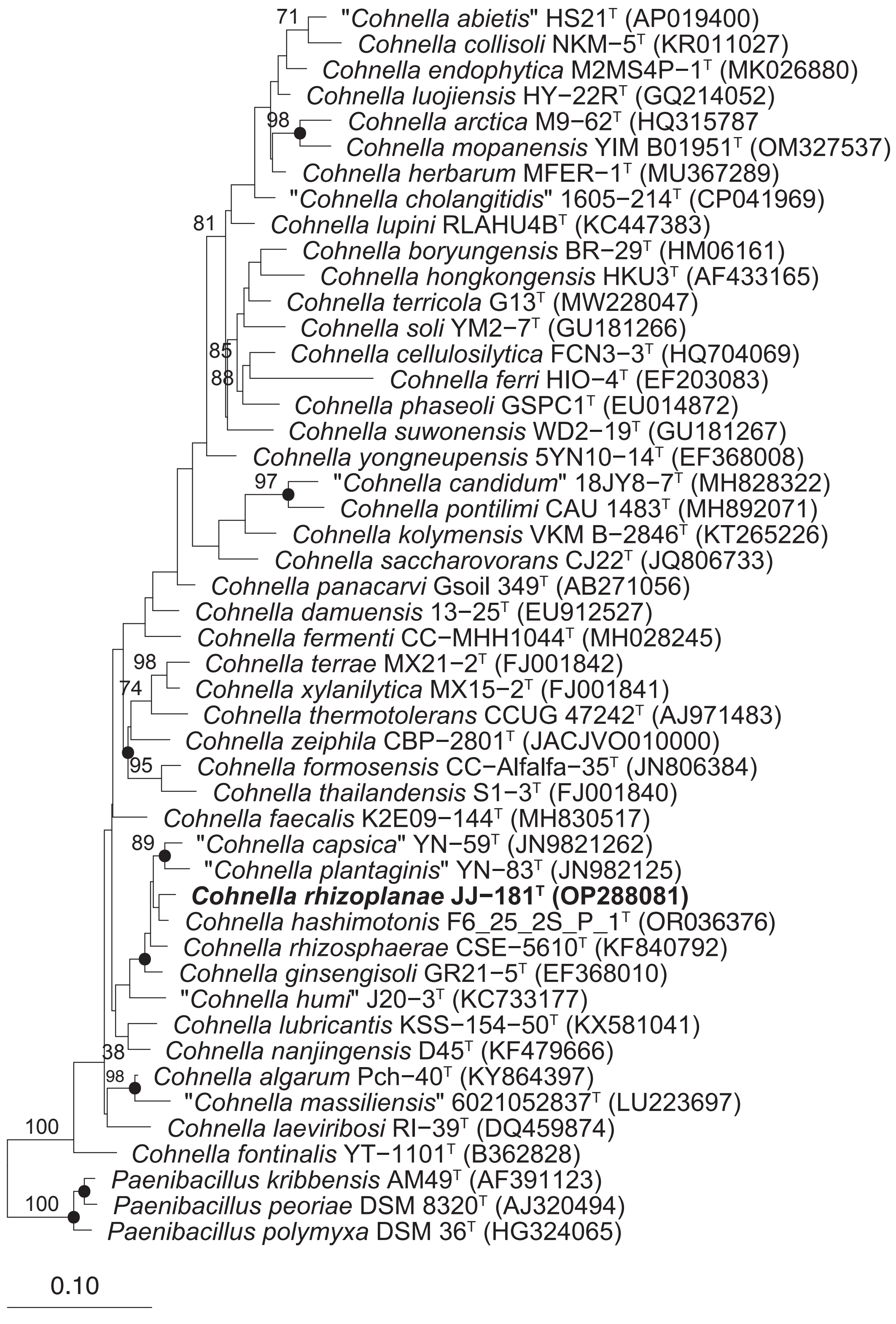

Figure S2. Whole-genome-based phylogenetic tree showing the phylogenetic relationship of strain JJ-181^{T} to species of the genus *Cohnella*. This minimum evolution tree was inferred using JolyTree (https://gitlab.pasteur.fr/GIPhy/JolyTree). The genomes of the type species of *Paenibacillus*, *Saccharibacillus* and *Xylanibacillus* were used as an outgroup. The genome sequence accession is specified between parentheses next after each taxon name. Branch supports were assessed by the rate of elementary quartets, as estimated by JolyTree (only supports > 0.5 were specified). Bar, 0.025 nucleotide substitutions per site.

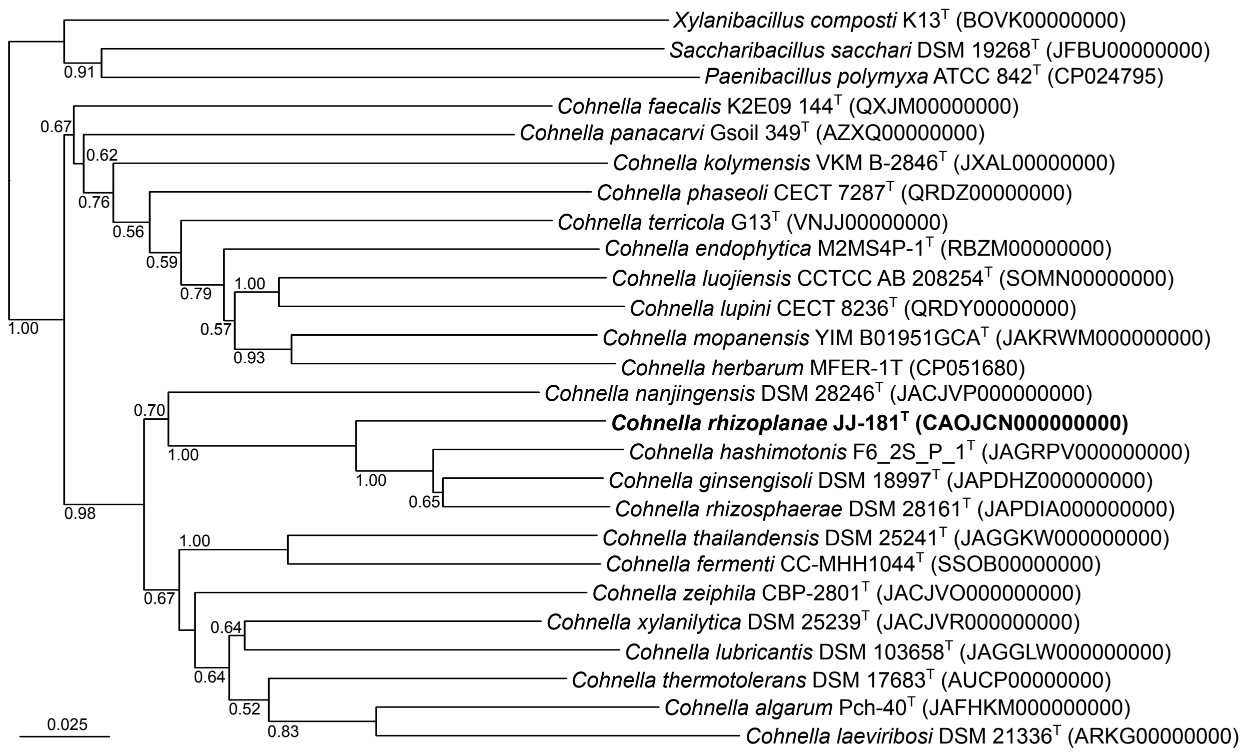

Figure S3. Number of CDS and distribution of the number of assigned COG categories for each *Cohnella* type strain. Assembly accession is specified between parentheses next after each taxon name. COG functions are detailed at the bottom.

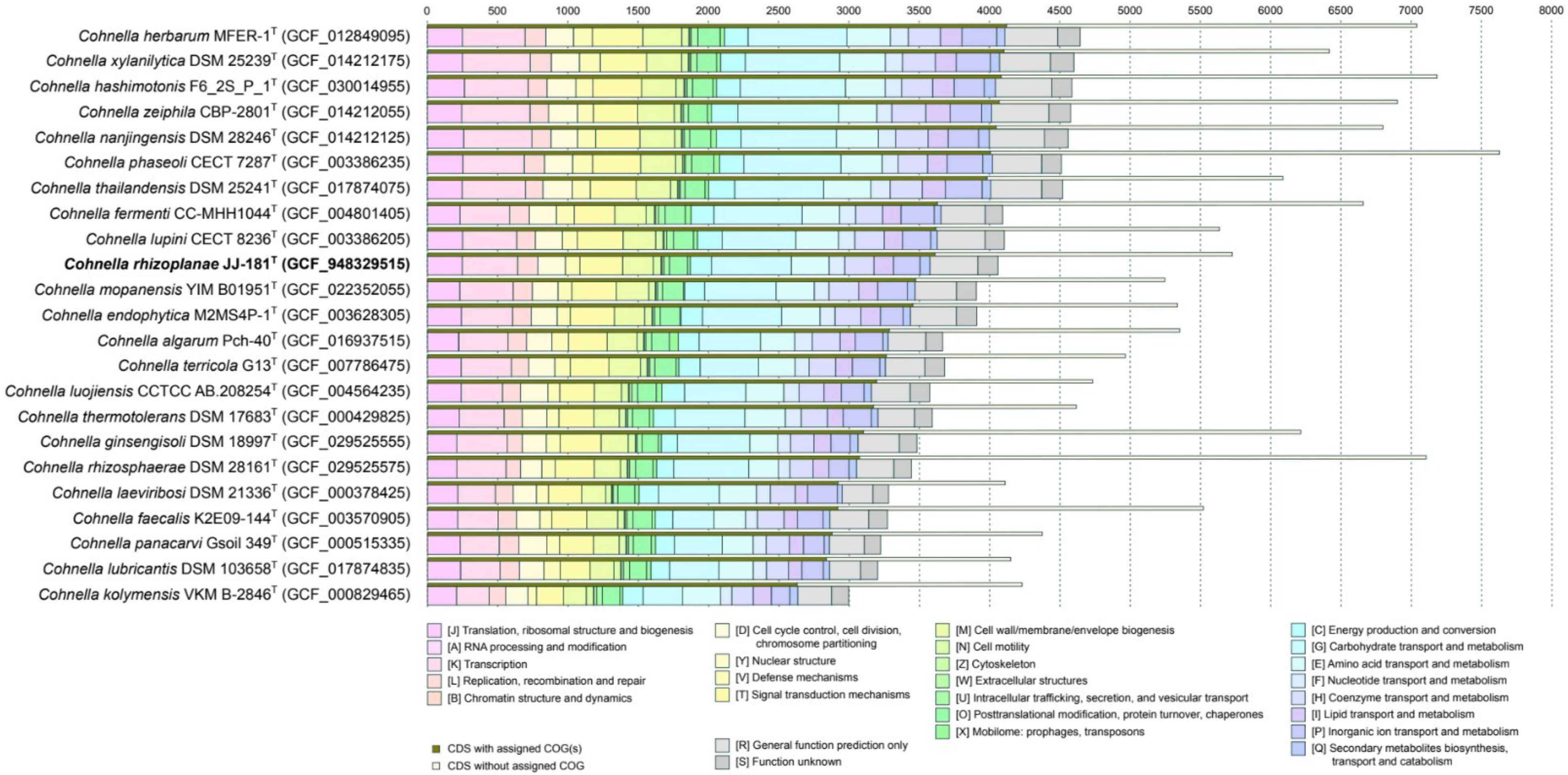

Figure S4. Polar lipid profile of strain JJ-181^T after staining with molybdatophosphoric acid.
Abbreviations: DPG, diphosphatidylglycerol, PG, phosphatidylglycerol; PE, phosphatidylethanolamine; PL2, unidentified phospholipid; APL1, 2, unidentified aminophospholipids; PL1, unidentified lipids only detectable with molybdatophosphoric acid.

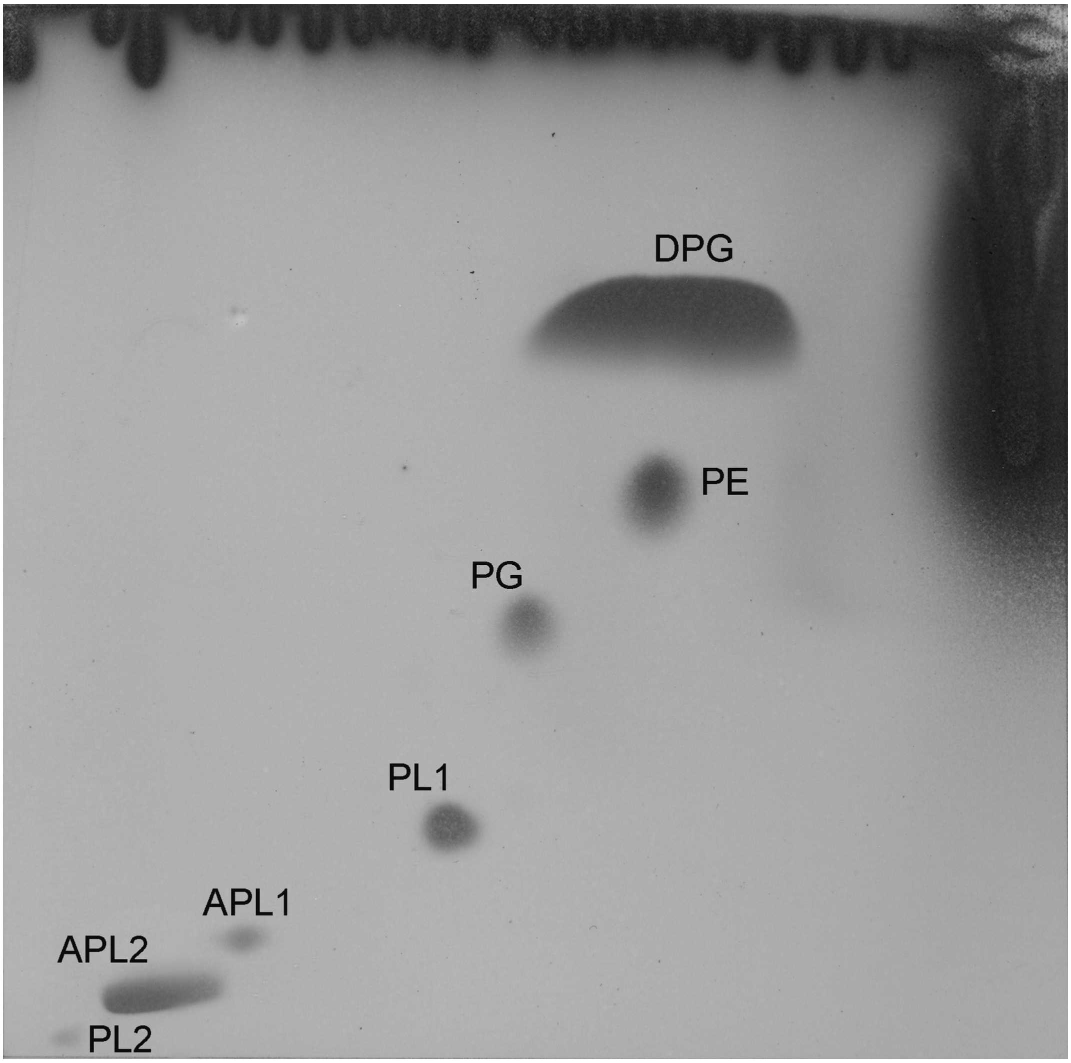

Supplement: Supplementary file 1 — Supplementary file1 (PDF 960 KB) [file 10482_2024_2051_MOESM1_ESM.pdf]
